# Effect of Probiotic Supplement on Cytokine Levels in HIV-Infected Individuals: A Preliminary Study

**DOI:** 10.3390/nu7105396

**Published:** 2015-09-28

**Authors:** Katia Falasca, Jacopo Vecchiet, Claudio Ucciferri, Marta Di Nicola, Chiara D’Angelo, Marcella Reale

**Affiliations:** 1 Clinic of Infectious Diseases, Department of Medicine and Science of Aging, University “G. d’Annunzio” Chieti-Pescara, 6610 Chieti, Italy; k.falasca@unich.it (K.F.); claudio.ucciferri@unimol.it (C.U.); 2 Laboratory of Biostatistics, Department of Medical, Oral and Biotechnological Sciences, University “G. d’Annunzio” Chieti-Pescara, 66100 Chieti, Italy; mdinicola@unich.it; 3 Unit of Immunodiagnostic and Molecular Pathology, Department of Medical, Oral and Biotechnological Sciences, University “G. d’Annunzio” Chieti-Pescara, 66100 Chieti, Italy; chiara_dangelo@hotmail.it (C.D.); mreale@unich.it (M.R.)

**Keywords:** *Lactobacillus casei* Shirota, dietary supplements, nutrition, AIDS, ART, inflammation

## Abstract

Inflammation persists in patients infected with HIV. Reduction of inflammatory cytokines and microbial translocation might be one way that this could be managed. Purpose: The anti-inflammatory properties of certain probiotic strains prompted us to investigate whether a probiotic could reduce the inflammatory index of HIV-infected patients. Methods: The study involved 30 HIV+ males on antiretroviral therapy, who were given one bottle of fermented milk Yakult Light^®^ containing Lactobacillus casei Shirota (LcS) twice a day for four weeks. Results: The probiotic LcS was associated with an increase of T lymphocytes and a significant increase of CD56+ cells (*p* = 0.04). There was also a significant decrease of mRNA levels of TGFβ, IL-10 and IL-12 (*p* < 0.001) and IL-1β expression (*p* < 0.001) and an increase of serum IL-23 (*p* = 0.03). In addition, decreased inflammation and cardiovascular risk were observed, as shown by a reduction of cystatin C (*p* < 0.001). Conclusions: These data provide preliminary evidence that probiotic supplementation may modulate certain immunological parameters and some of the cytokines that were analyzed. Thus, we propose that LcS may be an inexpensive and practical strategy to support the immune function of HIV+ patients.

## 1. Introduction

HIV infection causes high-level and long-term immune activation and inflammation-associated diseases. The idea that persistent immune activation and inflammation contribute to higher rates of non-AIDS comorbidities, such as cardiovascular, liver, kidney and neurologic diseases, is not new [1,2,3]. Yet, immune activation may also be seen as a normal and positive event following infection, and T cell activation levels may be predictive of prognosis in infected patients. The underlying mechanism of immune activation is poorly understood; however, multiple studies into its potential causes indicate that HIV replication below clinically-detectable levels might contribute to persistent immune activation [4]. Inflammation is a complex biological process involving an interplay of multiple cellular and inflammatory mediators that are affected by both HIV and antiretroviral therapy (ART) [5]. The accepted hypothesis is that the systemic inflammation observed in virologically-suppressed HIV-infected subjects is partially due to the breakdown of microorganisms in the gut mucosa, together with increased translocation of lipopolysaccharide (LPS; a component of the bacterial cell wall) and dysfunction of immunoregulatory cytokine production [6,7,8]. Microbial translocation is facilitated by HIV-induced depletion of CD4+ T cells from the gut-associated lymphoid tissue, and intestinal barrier dysfunction has been proposed as a potential cause of persistent immune activation [9,10]. Even after initiation of ART, microbial translocation does not normalize and continues to be associated with T cell activation [11]. However, ART intensification trials did not provide results that were sufficiently consistent to dismiss the role of HIV replication in persistent immune activation [12]. The majority of HIV-infected individuals is prone to coinfection with subsequent immune activation [13].

Recent studies have shown that probiotics may counteract the inflammatory process by stabilizing the gut microbial environment and the intestinal barrier, lowering systemic inflammation and stimulating natural killer (NK) cell activity. The mechanisms by which probiotics modulate the immune system, however, are not entirely understood [14,15]. It is well known that *Lactobacillus casei* Shirota (LcS), a commercial probiotic strain, increases the numbers of bacterial species in the gut that are considered beneficial, improves the balance between beneficial and potentially harmful intestinal bacteria and enhances NK cell activity [16,17,18,19]. Several *in vitro* studies have also shown that LcS enhances NK cell activity and induces IL-12 production in human peripheral blood mononuclear cells (PBMC) from healthy subjects. Furthermore, heat-killed LcS has been shown to stimulate IL-10, IL-12, TNF-α and IFN-γ production, to promote NK cell activity and to activate CD69 expression on NK cells [20]. It is still not fully understood, however, whether daily intake of LcS can modulate spontaneous production of cytokines in PBMC and contribute to the peripheral cytokine pool.

The objective of the current pilot study was to determine the effect of an LcS-fermented milk drink on the inflammatory state of HIV-infected patients on ART, by measuring serum immunoregulatory cytokines and their expression and production in freshly isolated PBMC.

## 2. Materials and Methods

### 2.1. Subjects

This was a single-center, open-label, prospective study that included thirty (30) Caucasian male subjects with HIV infection who were under continuous ART at the clinic of the Infectious Diseases, Department of Medicine and Science of Ageing, “G. d’Annunzio” University (Chieti-Pescara, Italy).

Patients were clinically stable and had a constant plasma viral load of <40 copies HIV RNA/mL and a CD4+ cell count of >300 cells/mL during the six-month period before the start of the study. The patients had not had any opportunistic infections during this time and had been on continual ART for 12 months before the study started. During their baseline visit, blood was taken for biochemical and hematological measurements. An interview was also conducted to obtain data on the subjects’ demographics and medical history. Clinical assessment included anthropometric measurements and physical examination. Subjects were encouraged throughout the study to report any adverse events or changes in their condition. Following the four-week period of LcS supplementation, subjects returned for a follow-up visit. A non-validated questionnaire prepared by the research team was administered to all subjects. This was used by the subjects to give a self-assessment of their general feeling of well-being, diet, social life and daily activities, plus compliance with regard to their consumption of the probiotic drink.

Patients excluded from participation were those: (1) using steroids, growth hormone, testosterone or any anabolic agent in the previous six months; (2) engaged in drug abuse; (3) with an acute infection in the previous three months; (4) with kidney disease and reduced glomerular filtration rate; (5) with hepatic disease; and (6) those treated with other food supplements before the beginning of the study. Participants were asked to adhere to their usual diet and lifestyle during the study.

### 2.2. Study Design

The study protocol was approved by the Ethics Committee at the University “G. d’Annunzio” Chieti-Pescara (Ethics Committee Project No. 13, 18 July 2013) and was performed in accordance with the ethical standards laid down in the 1964 Declaration of Helsinki. The volunteers were informed that the study was investigating the effects of the probiotic LcS on immune function; all subjects gave informed consent prior to their inclusion in the study. The subjects consumed one bottle of Yakult Light^®^ (a commercially-available probiotic fermented milk drink) containing a minimum of 6.5 × 10^9^ CFU LcS/bottle twice a day for four weeks. Overnight fasting venous blood samples were collected for immunological analyses at baseline and at the end of the intervention period for analysis.

### 2.3. Preparation of Serum Samples

Blood was collected in serum separator vacutainer tubes (BD Biosciences, Oxford, UK) and centrifuged at 3000 rpm for 10 min. Aliquots of serum were stored at −20 °C for later analysis.

### 2.4. Biochemical Analyses

Fasting venous blood samples were collected from the antecubital vein of all participants at their first clinic examination (T_0_) and after four weeks (T_4_). These were used to determine plasma levels of glucose, triglycerides, total cholesterol, high-density lipoprotein (HDL)-cholesterol, low-density lipoprotein (LDL)-cholesterol, aspartate aminotransferase (AST), alanine aminotransferase (ALT), C-reactive protein (CRP), erythrocyte sedimentation rate (ESR), creatinine, urea nitrogen, serum cystatin C and microalbuminuria. Routine laboratory tests were performed at the Division of Clinical Pathology in the same hospital.

### 2.5. Virologic and Immunologic Markers

CD4± and CD8± T cell counts were obtained by flow cytometry of lymphocyte subpopulations. Plasma viral load (HIV-RNA) was determined using the “Amplicor” method (Roche Molecular Diagnostics, Milan, Italy) with a detection limit of >40 HIV RNA copies/mL of plasma.

### 2.6. Preparation of PBMC

Fasting blood samples were taken from the volunteers in the morning between 08:00 and 10:00 in vacutainer tubes containing sodium heparin. Ten milliliters of blood/saline (1:2 v/v) were layered over 5 mL of Ficoll-Paque and centrifuged at 1600 rpm for 30 min at room temperature. Cells were harvested from the interface, resuspended in RPMI 1640 medium and washed twice. These steps were repeated to achieve a lower degree of erythrocyte contamination. Cell concentration was adjusted to 2 × 10^6^ cells/mL and incubated for 24 h at 37 °C, 5% CO_2_, in RPMI 1640 medium supplemented with 10% fetal calf serum, 4 mM l-glutamine, 25 mM HEPES buffer, 50 U/mL penicillin and 50 mg/mL streptomycin (all media and components were purchased from SIGMA, Italy). At the end of incubation, supernatants were collected and stored at −20 °C for later analysis of spontaneous cytokine production.

### 2.7. mRNA Extraction and Reverse Transcription-Polymerase Chain Reaction Analysis

Total RNA was extracted from PBMC isolated from HIV-infected patients using TRIzol Reagent (Invitrogen, Life Technologies, Paisley, UK), according to the manufacturer’s protocol. The RNA concentration and purity were determined using a NanoDrop ND-1000 spectrophotometer (NanoDrop Technologies, Wilmington, DE, USA). RNA purity was assessed by using the Agilent 2100 Bioanalyzer (Agilent Technologies, Santa Clara, CA, USA). RNA samples were kept frozen at −80 °C until use. Purified RNA was electrophoresed on a 1% agarose gel to assess the integrity of the purified RNA. One microgram of RNA was reverse transcribed into cDNA using a High Fidelity Superscript reverse transcriptase kit (Applied Biosystems, Foster City, CA, USA) in accordance with the manufacturer’s instructions. mRNA/cDNA specific cytokine primer pairs were designed, and polymerase chain reaction (PCR) was performed using the specific primer pairs in PCR-express cyclers (Hybaid, Heidelberg, Germany): IL-1β forward 5′-TGAGGATGACTTGTTCTTTGAAG-3′, reverse 5′-GTGGTGGTCGGAGATTCG-3′; IL-10 forward 5′-GAGAACCAAGACCCAGACATC-3′, reverse 5′-TCACTCATGGCTTTGTAGATGC-3′; IL-4 forward 5′-CAAGTGACTGACAATCTGGTG-3′, reverse 5′-AGTGACAATGTGAGGCAATTAG-3′; IL-12/23p40 forward 5′-TATGTCGTAGAATTGGATTGG-3′, reverse 5′-AAACTCTTTGACTTGGATGG-3′; TGFβ forward 5′-AACAATTCCTGGCGATACCTC-3′, reverse 5′-GTAGTGAACCCGTTGATGTCC-3′; IL-17A forward 5′-CAACGATGACTCCTGGGAAG-3′, reverse 5′-GGGATTGGTATTGGTATTCCGG-3′; 18S forward 5′-CTTTGCCATCACTGCCATTAAG-3′, reverse 5′-TCCATCCTTTACATCCTTCTGTC-3′. The appropriate number of cycles was pre-established for every set of samples to ensure that amplification was in the exponential phase of the PCR. PCR products were separated by gel electrophoresis on 2% agarose gels and visualized by ethidium bromide staining. All gels were scanned, and the normalized intensities of all reverse transcription (RT)-PCR products were determined by the Bio-Rad gel documentation system (Bio-Rad, Hercules, CA, USA). Mean and standard deviation intensities were calculated for all PCR experiments.

### 2.8. Cytokine Measurements

Human cytokine levels in serum and in cell-free supernatants of PBMC from HIV-infected and ART-treated patients were quantified using specific enzyme-linked immunosorbent (ELISA) assays. ELISA assays were conducted with commercial kits (Endogen, Woburn, MA, USA) according to the manufacturer’s instructions. The plates were read at 450 nm, and the absorbances were transformed to pg/mL, using calibration curves prepared with cytokine standards included in the kits. The intra- and inter-assay reproducibilities were >90%. Duplicate values that differed from the mean by greater than 10% were not considered for further analysis.

### 2.9. Statistical Analysis

Sample size determination was based on the main endpoint, the reduction of IL-1β following 4 weeks of treatment (T_4_). Assuming a difference in variation of at least 2% in IL-1β between T_0_ and T_4_ levels, using a *t*-test for unpaired data at a level of 0.05 with 80% power and the common standard deviation of 2.5 pg/mL, at least 30 patients were needed. This evaluation was performed using the “sample size” function of R 3.0.2 open source software.

Qualitative variables were summarized as the frequency and percentage and quantitative variables as the mean and standard deviation (SD) or standard error (SE). The Shapiro–Wilk test was performed to detect departures from the normal distribution. The Wilcoxon U-test for paired data was applied to compare the levels of the quantitative variables between T_0_ and T_4_.

Univariate linear regression was performed to analyze the association between the variation of cytokine levels and the baseline characteristics of patients.

Two-tailed *p*-values were calculated for all tests, and a *p*-value of 0.05 or less was considered statistically significant. All statistical analyses were performed using SPSS^®^ software 11.0 (SPSS Inc., Chicago, IL, USA).

## 3. Results

### 3.1. Study Population

Demographic data and the clinical characteristics of patients at baseline are reported in Table 1. All enrolled patients completed the study. There was 100% compliance with the dietary intervention.

**Table 1:** Demographics data and clinical characteristics of patients at baseline.

Patients characteristics	
Age (year), mean ± SD	42.3 ± 11.3
CDC stage, *n* (%)	
A	20 (66.7)
B	8 (26.7)
C	2 (6.6)
Risk factor, *n* (%)	
Heterosexual	12 (40.0)
Homosexual	11 (36.7)
IDU	7 (23.3)
Time from diagnosis (months), median (IQR)	69 (42–110)
Time of treatment (months), median (IQR)	47 (13–87)

CDC: Centers for Disease Control and Prevention; IDU: injection drug users.

All patients infected with HIV were treated with combined ART according to currently accepted guidelines. Their viral load was stable and undetectable before the start of LcS intake (T_0_) and after four weeks of LcS intake (T_4_). The addition of LcS to their daily diet was well tolerated by all of the patients; no adverse symptoms were reported (fever, nausea or stomach pain).

The questionnaire showed that most patients (94.1%) were generally satisfied and found the LcS-fermented milk drink acceptable.

### 3.2. Effects on Blood Metabolic Markers

No significant differences were found in the mean serum concentrations of principal metabolic parameters, particularly in glucidic and lipidic assessments. Indices of liver and kidney functions did not show any statistically-significant change. Furthermore, the principal inflammatory markers ESR and CRP did not show any statistically-significant differences during this study (data not shown). Cystatin C serum levels, however, were significantly reduced after the LcS intake (0.75 ± 0.03 *vs.* 0.72 ± 0.03 mg/L with *p* < 0.001).

### 3.3. Effects of LcS on Circulating T Lymphocyte Subtype Counts

**Figure 1:**
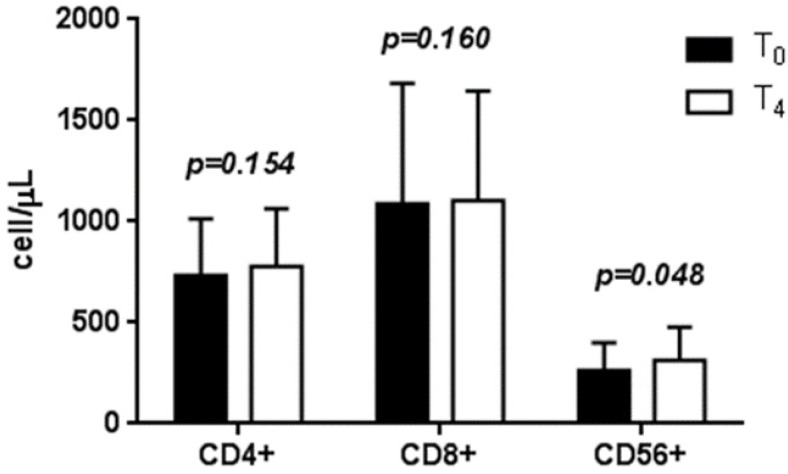
Mean ± standard error of T lymphocyte subtype counts.

Comparison of baseline measurements to those taken at the four-week follow-up showed that the CD4+ cell count increased on average by 45.9 ± 35.2 cells/μL (732.2 ± 208.1 *vs.* 778.1 ± 286.8 cell/µL, *p* = 0.154). The CD8+ cell count increased on average by 16.4 ± 49.3 cells/μL (1087.8 ± 694.5 *vs.* 1104.2 ± 642.2 cell/µL, *p* = 0.160). The CD56+ cell count showed a significant increase of 48.7 ± 21.7 cells/μL (263.4 ± 137.6 *vs.* 312.1 ± 167.3 cell/µL, *p* = 0.048) (Figure 1). No significant variation was observed in the CD4+/CD8+ ratio.

### 3.4. Serum Cytokine Levels before and after LcS Intake

Most of the cytokines analyzed in the serum of HIV-infected patients after four weeks of LcS intake did not show significant differences, except for IL-23, which showed a statistically-significant increase (*p* = 0.037). A trend to lower levels of TGFβ, IL-10 and IL-12 was observed, as well as a trend for higher levels of IL-4, but these data were not statistically significant (Table 2). While serum samples from most of the ART-treated patients contained measurable amounts of the selected cytokines, only a few patients showed measurable IL-1β levels.

**Table 2:** Serum levels of cytokines.

Cytokines (pg/mL)	T_0_	T_4_	(T_4_-T_0_)	*p*-value ^a^
TGF-β	4162.1 ± 2325.9	1345.4 ± 290.5	−2816.7 ± 2097.5	*0.417*
IL-23	9.0 ± 0.7	9.8 ± 1.1	0.8 ± 1.5	*0.037*
IL-4	3.6 ± 1.0	5.6 ± 2.1	1.6 ± 1.9	*0.433*
IL-10	4.7 ± 1.9	4.4 ± 0.9	−0.4 ± 1.7	*0.619*
IL-12	938.1 ± 259.6	874.1 ± 233.3	−44.5 ± 75.5	*0.104*

Mean ± standard error of the serum levels of cytokines. ^a^ Wilcoxon U-test for paired data T_0_ *vs.* T_4_ levels.

### 3.5. Effects on Cytokine Expression

**Figure 2:**
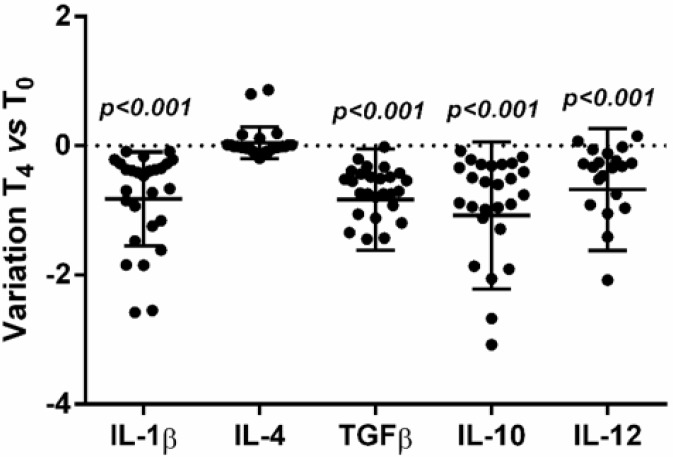
Variation of cytokine mRNA expression after 4 weeks of LcS intake respect to baseline level.

The source of cytokine production during HIV infection is only partly understood. In the very early phase of HIV infection, it is likely that cytokines are produced locally, whereas, after virus dissemination, they are produced at more general and distant sites. To evaluate whether the pattern of serum findings could be related to production by PBMC, we evaluated the spontaneous expression of selected cytokines in unstimulated PBMC isolated from HIV-infected subjects, before and after LcS intake. Gene expression of the cytokines measured in serum after four weeks of LcS intake showed changes. Significantly decreased mRNA levels of TGFβ, IL-10 and IL-12 (*p* < 0.001) and increased (although not statistically significant) levels of IL-4 mRNA (*p* = 0.330) were observed (Figure 2). LcS intake was associated with significantly reduced IL-1β expression (*p* < 0.001).

### 3.6. Effects of LcS on Cytokine Production by PBMC

In order to evaluate whether LcS may also control cytokine production by a post-transcriptional mechanism, we analyzed the spontaneous cytokine production in PBMC isolated from HIV-infected patients before LcS intake (T_0_) and after four weeks of LcS intake (T_4_). At T_0_, spontaneous production of cytokines by PBMC was very low. At T_4_, we observed a statistically-significant increase of TGF-β (*p* = 0.004), a non-statistically-significant increase of IL-10 and IL-4 and a decrease of IL-23 (Table 3). Univariate linear regression analysis showed that variation in T lymphocyte subtype counts and the variation of cytokine levels were not statistically-significantly associated with any baseline characteristic of the patients.

**Table 3:** Cytokine levels in the supernatants of non-stimulated PBMC.

Cytokines (pg/mL)	T_0_	T_4_	(T_4_-T_0_)	*p*-value ^a^
TGF-β	25,042.2 ± 1614.2	25,984.0 ± 1924.6	941.8 ± 1438.2	*0.004*
IL-23	11.5 ± 0.8	10.1 ± 1.0	−1.4 ± 0.6	*0.989*
IL-4	33.4 ± 25.1	35.6 ± 25.3	2.2 ± 2.4	*0.398*
IL-10	14.4 ± 6.5	16.6 ± 5.7	2.2 ± 4.5	*0.424*

Mean ± standard error of cytokine levels in the supernatants of non-stimulated PBMC. ^a^ Wilcoxon *U*-test for paired data T_0_ *vs*. T_4_ levels.

### 3.7. Th1/Th2 Ratio

Th1/Th2 bias was assessed by examining the ratio of IL-4 or IL-10 Th2 cytokines to proinflammatory IL-1 or IL-12 Th1 cytokines. After four weeks of LcS intake, the IL-4/IL-1β mRNA expression ratio showed a significant increase of 0.41 ± 0.10 (*p* = 0.002). The IL-10/IL-12 and IL4/IL-12 ratios were not statistically significantly modified after four weeks of LcS intake (*p* = 0.062 and *p* = 0.433, respectively).

## 4. Discussion

The main findings of this study of HIV-infected patients on ART was that consumption of LcS-fermented milk drink was associated with an increase of T lymphocyte subtype count and a reduction of inflammatory status, as assessed by serum levels of immunoregulatory cytokines and their expression and production in freshly isolated PBMC.

Cytokines are short-lived proteins; their effects are temporary unless there is a signal for more to be produced. Some cytokines promote inflammation, while other cytokines act to dampen it. Both proinflammatory and regulatory cytokines contribute respectively to tissue pathology and transcription of latent HIV-1 or to the induction of polyclonal B-cell activation, so their role during HIV infection and ART has been studied by various working groups, but with conflicting results [21,22,23,24,25].

Even though the benefits of ART for HIV+ persons are well documented, it has been consistently observed that elevated levels of inflammation, immune activation and immune dysfunction persist in ART-treated individuals, despite successful suppression of plasma viremia [26]. Non-AIDS comorbidities, such as cardiovascular disease [2,27,28,29], neurocognitive impairment [30], diabetes mellitus [31,32], osteoporosis [33] and malignancies, are associated with an elevated activity of inflammatory cell and chronic immune activation. This may also contribute to increased mortality during ART treatment. With ART, the virus load can be controlled for prolonged periods, but it cannot be eliminated; continued replication of the virus induces chronic antigenic stimulation and immune hyperactivation.

A breakdown in the gut mucosa together with microbial translocation play a central role in disease pathogenesis and the persistent inflammation observed in HIV+ patients and also contribute to fibrosis in lymphoid tissues [34]. It is well documented that probiotic bacteria are associated with a range of health benefits. Numerous studies investigating their influence on the gut microbiome and mucosa have been conducted; prebiotics have also been investigated with some early positive results. The immunomodulatory effects of probiotics are strain dependent. For example, several studies have reported no effect of probiotics on the *in vivo* and *in vitro* production of cytokines, whilst other studies have reported that a mixture of probiotic strains enhanced the production of the anti-inflammatory cytokines, but had no or a slight effect on proinflammatory cytokines [35,36]. Other studies have reported that the addition of *Lactobacillus casei* to PBMC cultures induced cytokine production, but inhibited LPS-induced cytokine production when added together with LPS [37,38,39]. There are also reports of a lack of effect of probiotics on cell proliferation and cytokine release in mesenteric lymph nodes in Wistar rats [40]. Thus, the effects on the cytokine profiles of each individual probiotic strain should be established. To have beneficial effects, probiotics need to reach the small intestine and transiently colonize the host. Since LcS is known to be able to survive gastric transit, we evaluated the effect of daily LcS consumption on the cytokine network in HIV-infected patients.

TGF-β is a cytokine that contributes to the immune suppression observed in HIV-infected individuals [41]. Thus, we evaluated the effect of daily intake of LcS on TGF-β: a reduction of serum levels and spontaneous mRNA expression was observed. Surprisingly, levels of TGF-β released by PBMC in culture were also significantly increased. These observations might be due to the rapid modulation of gene expression, possible high cytokine concentrations at the site of release and much lower concentrations after their dilution in blood [42,43].

A pathogenic role for IL-10 in HIV infection was suggested by a study that showed that the viral antigen drove the increase of IL-10 levels that contribute to a reversible T cell dysfunction in HIV-infected persons. ART induces a significant, gradual decrease in IL-10 levels, but without their normalization. The inhibition of the IL-10 pathway significantly increases HIV-specific CD4+ and CD8+ T cell proliferation and effector T cell functions [44,45,46]. We observed significantly reduced mRNA expression and a weak decrease of IL-10 levels in the serum of HIV-infected subjects who consumed LcS. Based on this finding, we suggest that probiotic supplementation for a longer time and in addition to ART may help to downregulate IL-10 and, thus, improve the management of HIV+ patients under ART.

In HIV+ patients who exhibit increased CD4+ T cell counts during ART, it has been shown that the ability of PBMC to produce IL-23 is reduced, which compromises the immune response to opportunistic infections [47,48]. We investigated if LcS intake for a four-week period could influence levels of IL-23 in our patients. Our results showed a significant increase of IL-23 levels in serum, but there was no concomitant release of IL-23 by PBMC in culture, probably because cells other than PBMC contribute to circulating IL-23 levels. IL-12 and IL-23 are related cytokines that share the p40 subunit of IL-12. As p40 can associate with p35 to form IL-12p70 or with p19 to form IL-23, a comparison with these bioactive cytokines will be necessary. In cells infected *in vitro* with HIV, impaired IL-12 production has been observed [49], and in PBMC from chronically HIV-infected patients, IL-12p70 production has been shown to be dependent on different *in vitro* stimuli [50]. The present study showed that LcS intake did not significantly modulate serum levels of IL-12 in patients. The reduced spontaneous IL-12p35 mRNA expression may drive the increase of p40 production in favor of IL-23 production rather than IL-12 production. We suggest that LcS intake could drive the balance in favor of IL-23 over IL-12 and that potential restoration of IL-12 responses might be expected from a longer period of LcS supplementation (together with ART). IL-23 preferentially stimulates proliferation of memory Th1, maintaining a Th1-committed memory response [51], and IL-23 signaling is completely absent in Th17 cells from HIV-infected individuals.

HIV-infected patients have significantly low levels of Th17 cells that are not restored by ART [52]. A recent study suggested that probiotics may enhance gastrointestinal immunity and, thus, lead to the restoration of Th17 cells in the mucosa [53]. The significant increases of IL-23 and reduced LPS levels observed in the serum of HIV-infected patients after four weeks of LcS consumption encouraged us to study the effect of LcS on circulating IL-17 levels. A wide overlap between IL-17 serum levels detected before and after LcS intake was observed. The differences did not reach the level of significance in our patients, indicating that further research is warranted. Human trials with a longer period of LcS supplementation are needed to confirm the LcS immunomodulatory effects *in vivo*.

It is known that IL-12 and IL-10 display apparently opposing roles in the regulation of the Th1 immune response [54]. This is why several studies have used the IL-10/IL-12 ratio to assess immune status, *i.e.*, as a measure of the balance between anti-inflammatory and proinflammatory states [55]. In our patients, the IL-10/IL-12 ratio was not statistically significantly modified after four weeks of LcS intake, suggesting that LcS does not have a Th2/Th1-specific downregulatory function. A previous study had shown that ART suppresses spontaneous over-secretion of cytokines, such as IL-1β [56]. To evaluate if LcS intake might improve this ART effect, we analyzed the levels of IL-1β in the patients given the LcS-fermented milk drink, but were not able to fully demonstrate an LcS immunomodulatory effect. In fact, while mRNA IL-1β expression was significantly reduced, no significant differences were found in serum levels, which were close to the detection limit. We suggest that the cytokine levels detected in circulation may not be a true representation of the actual levels in local microenvironments, where complex cytokine interactions may occur that could induce or reduce cytokines.

A recent study has shown that IL-4 acts rapidly through a Stat6-dependent and IL-12-independent mechanism to induce NK and NKT cells to produce IFN-γ [57]. Thus, the increased IL-4 levels in ART HIV-infected patients that received for four weeks LcS may represent a potential mechanism for enhancement of NK activity that warrants further investigation. The selective depletion of CD56+ T cells shown in HIV-infected individuals could be involved in the decreased peripheral blood T cell cytotoxicity found in HIV infection. Thus, in the HIV-infected patients given LcS, the observed significant increase of CD56+ cells together with a weak increase of IL-4 is very interesting. This result is in accordance with other studies showing that CD56+ cells are capable of the secretion of cytokines, including IL-4 [58], acting as a versatile population whose functional capacities are determined by cytokines present in their microenvironment [59,60].

LcS supplementation in the HIV-infected patients seemed to have no direct effect on HIV replication and did not interfere with ART, but there was an increase, albeit not significant, of CD4+ lymphocytes. In addition, the LcS supplementation did not interfere with any metabolic, renal and hepatic functions. Finally, our study showed a decrease of cystatin C after LcS supplementation. Data about cystatin C in HIV-infected patients are limited to previous studies from selected cohorts, showing elevated cystatin C levels in HIV+ patients with impaired kidney function [61], but it is clear that cystatin C is also an independent marker of atherosclerotic disease in the general population and HIV-infected patients [29]. Therefore, one might speculate that the reduction of cystatin C after intake of LcS could be linked to a reduction in cardiovascular risk [62].

Our results are partly in agreement with other *in vitro* and *ex vivo* studies, although such studies may not necessarily recreate conditions found *in vivo*. Complex and discordant results in probiotic research may be due to various factors, including differences in the probiotic strains or doses administered, the diversity of mammalian species investigated, as well as differences in the patient populations and stages of the disease investigated. It is important to put the present study into context. These are preliminary findings from an exploratory study involving a small number of patients given a short duration of LcS supplementation; there was also no control group of uninfected subjects. Our findings, however, are of sufficient interest to encourage further investigation in a larger cohort of patients, in order to determine whether changes in levels of a few cytokines, as we have observed in this small cohort, may modulate more cytokines to create a complicated regulatory circuit and eventual improved clinical outcome.

## 5. Conclusions

Our data show that administration of the LcS probiotic to viro-immunologically-stable HIV+ patients is safe and well tolerated. Our study has provided pivotal evidence that supplementation with this probiotic could be an affordable and proactive strategy to help HIV patients maintain a better immune response, through improvement of certain immunological parameters and reduction in key inflammatory cytokine markers. Although this type of evaluation, involving the stimulation of blood cell immune response by probiotics, does not represent the actual physiological reality, it is a practical test and a viable option to be used in exploring evidence of potential benefit, especially in patients with persistent and long-lasting disease.
